# Coupled Immunological and Biomechanical Model of Emphysema Progression

**DOI:** 10.3389/fphys.2018.00388

**Published:** 2018-04-19

**Authors:** Mario Ceresa, Andy L. Olivares, Jérôme Noailly, Miguel A. González Ballester

**Affiliations:** ^1^BCN-Medtech, Department of Information and Communication Technologies, Universitat Pompeu Fabra, Barcelona, Spain; ^2^ICREA, Barcelona, Spain

**Keywords:** COPD, emphysema, chronic bronchitis, finite element methods, agent-based models, biophysical modeling, multiscale modeling, supercomputing

## Abstract

Chronic Obstructive Pulmonary Disease (COPD) is a disabling respiratory pathology, with a high prevalence and a significant economic and social cost. It is characterized by different clinical phenotypes with different risk profiles. Detecting the correct phenotype, especially for the emphysema subtype, and predicting the risk of major exacerbations are key elements in order to deliver more effective treatments. However, emphysema onset and progression are influenced by a complex interaction between the immune system and the mechanical properties of biological tissue. The former causes chronic inflammation and tissue remodeling. The latter influences the effective resistance or appropriate mechanical response of the lung tissue to repeated breathing cycles. In this work we present a multi-scale model of both aspects, coupling Finite Element (FE) and Agent Based (AB) techniques that we would like to use to predict the onset and progression of emphysema in patients. The AB part is based on existing biological models of inflammation and immunological response as a set of coupled non-linear differential equations. The FE part simulates the biomechanical effects of repeated strain on the biological tissue. We devise a strategy to couple the discrete biological model at the molecular /cellular level and the biomechanical finite element simulations at the tissue level. We tested our implementation on a public emphysema image database and found that it can indeed simulate the evolution of clinical image biomarkers during disease progression.

## Introduction

Chronic obstructive pulmonary disease (COPD) is estimated to affect more than 500 million people worldwide, causing significant disability, loss of quality of life and social burden, with costs in excess of € 56 billion per year in the European Union (Decramer et al., 2012). The disease has a lifetime prevalence of about 28% and cigarette smoking is commonly considered to be the principal risk factor (Gershon et al., 2011). Recent projections suggest that COPD will be the third cause of global mortality by the year 2030.

The pathogenesis of COPD is still not completely understood (Larsson, 2007; Yoshida and Tuder, 2007) and involves a number of multi-scale cellular processes, including airways inflammation, adaptation and innate immunity to cigarette smoking, sensitivity to self and not-self antigens, accelerated senescence, and deregulation of mechanisms of cell repair (Repapi, 2010; Pavord et al., 2012). Interactions between the environment and a selected group of candidate genes is also considered very important (Akinbami et al., 2012; Mizuno et al., 2017; Zhao et al., 2017).

Clinical management of COPD involves consistent use of inhaled corticosteroids that help reducing COPD mortality. However, their efficacy is limited (Faner and Agustí, 2016) and many patients experience exacerbations and poor symptoms control (Brightling et al., 2012).

As a matter of fact, the clinic presentation of COPD is not homogeneous, but presents two main clinical phenotypes, emphysema and chronic bronquitis, each with many sub-types, different comorbidities and risk profiles (Martinez et al., 2012). Even if there is no therapeutic target that can reverse the decline of lung function over time (Vestbo et al., 2013), a **broader recognition** of markers associated with adverse risk (Partridge et al., 2006) and therapies that specifically target **different phenotypes** specifically reduce exacerbations and improve patient's life (Castro et al., 2010; Holgate, 2012).

An additional problem is that it is extremely challenging to do an early detection and staging of COPD. This is because the gold standard for clinical diagnosis is Pulmonary Function Tests (PFTs) which is not sensitive enough to detect any disease progression before a large part of the lung has been compromised (Cooper et al., 2017). It is also not sensitive enough to detect different subtypes and elucidate different mechanisms of actions.

Specifically in **emphysema**, the continued inflammation of lung parenchyma eventually leads to a loss of collagen and elastin in the alveoli (Sharafkhaneh et al., 2008; Goldklang and Stockley, 2016). As a result of this sustained damage the septa become increasingly compliant and eventually fail mechanically during normal breathing. This reduces the area available for gas exchange causing dyspnea and shortness of breath. In addition, the mechanical damage due to emphysema is likely to stimulate tissue repair mechanisms at cellular level, that result in the production of type I collagen (Crosby and Waters, 2010). As a matter of fact, alveolar fibrosis is observed in emphysematous spaces, in the form of thickened and stiffened alveoli, which most likely contributes to shortness of breath (Yousem, 2006).

Faced by this complexity in the mechanisms and the lack of a simple clinical tests, it is important to assess the patient by integrating information from heterogeneous sources such as molecular data and medical imaging, in order to adapt the treatment options with the phenotype and risk profile. A promising option is to include information from **computational models** of biological systems that can account for causative effects, otherwise difficult to apprehend in clinics. These models have the potential to predict complex behaviors, elucidate regulatory mechanisms, and inform experimental designs to eventually point out specific factors to control or therapeutic targets, in order to improve patient management (Di Ventura et al., 2006).

Cancer research has already exploited computational models over different spatial and temporal scales as **a promising way to describe complex diseases** (Deisboeck et al., 2009, 2011; Wang et al., 2015). There, multiscale models interact with clinical data to generate and test different hypotheses, facilitating drug development (Clancy et al., 2016) and optimizing delivery and therapeutic effect (Cristini et al., 2017). We refer the interested reader to the detailed review by Wang and Maini (2017).

Recent interdisciplinary advances contributed to unravel the complex pathophysiological mechanisms that occur in COPD on both the *macroscopic* and *microscopic* scale. In case of **macroscopic model of the respiratory system**, for example Bordas et al. (2015) describes how to obtain a specific mesh of the patient for CFD simulations and Berger et al. (2016) discuss the application of a poroelastic deformation model for pulmonary ventilation. Chernyavsky et al. (2014) proposes a theoretical model of the possible effect of inflammation on the restriction of small airways. The reader can also refer to the review of COPD multi-scale modeling by Burrowes et al. (2013).

Among others, the “Protective Artificial Respiration” initiative fundamentally contributed to the understanding of COPD. We would like to cite Wiechert et al. (2011) for their multiscale model of respiratory system that coupled large bronchi and small alveoli, as well as Roth et al. (2017a,b) respectively for a study of the essential interactions between flow and deformation in the lungs and a simplified model of lung microstructures. Also Verdugo et al. (2017) reported on efficient solvers for respiratory mechanics. Among the works devoted to particle deposition we recall the work of Freitas and Schröder (2008) for a numerical study of 3D flows in a human lung model, and Lintermann and Schröder (2017) for the simulation of aerosol particle deposition and Calmet et al. (2016) for their model and simulations of particle deposition based on High-Performance Computing. Very recently, an experimental characterization of the nonlinear compressible behavior of the parenchyma is reported in Birzle et al. (2018).

For the **microscopic modeling**, literature contains numerous works on the modeling of the immune system at the molecular level. For instance, Folcik et al. (2007) developed an agent-based model for the innate and adaptive immune system while (An, 2008) contributed an agent-based model of the epithelium. A model of inflammation with interactions between macrophages and fibroblasts capable of simulating scarring, tissue damage and fibrosis is presented in Brown et al. (2011). Most of the studies on AB modeling of COPD focus on emphysema, and mainly study the resulting destruction of the tissue. The most common method uses a 2D network of springs to represent alveolar tissue (Mishima et al., 1999). These modeling studies have the merit to highlight the redistribution of forces within the tissue during the progression of emphysema. This simulated progression was found to produce experimentally observed emphysema patterns (Suki et al., 2003) and was extended to 3D by Parameswaran et al. (2011) through the use of cuboidal cells to represent the alveoli. The European AirProm project has initiated the study of multi-scale models for the study of COPD (Burrowes et al., 2013).

In the INSPIRE project^1^, we would like to give a multi-scale, multi-physics description of the phenomena that cause the onset of emphysema and the possibility to predict the risk profile of the patient. Accordingly, the **main purpose** of the **presented work** is to propose a multi-scale model, able to integrate known interactions among inflammation, remodeling and parenchyma destruction, with particular attention to the role played by the immune system. We extended our previous work Ceresa et al. (2017) to couple the dynamics of the biological events captured through agent cooperation in an agent-based (AB) model with a biomechanical simulation of the tissue captured by a coupled Finite Element (FE) Model that iteratively predicts the evolution of the mechanical cues transmitted to the cells inside the lungs. We hope that such model could, once properly refined and validated, add to the interpretation of the specific disease phenotype toward the prediction of personalized risk profiles. We think our model builds nicely on the previous cited literature for the microscopic models because we use a less simplified model of the molecular interactions. In addition, we explicitly take into account the mechanical forces the tissue is subjected to using a well-vetted FE model, while others have worked more on connection models with elastic spring.

In the following sections we will discuss the coupled AB and FE model that we contribute (section Methods), and the experimental setup designed to validate the model on a public CT dataset of emphysema images (section Experimental Setup). We then present the results of the experiments, their discussion (section Results and Discussion) and the conclusions and future works (section Conclusions and Future Works).

## Methods

As we commented before, research and clinical practice suggest that emphysema development happens along two different time-scales: a slow molecular one due to the inflammatory response to solid particles (Cosio et al., 2009), and a rapid one, caused by sudden rupture of the alveolar walls due to mechanical forces which act on lung tissue during respiration (Suki et al., 2003).

In the following sections, first we present a dynamic model of inflammatory response using ordinary differential equations (ODE) taken from literature that does not account for spatial and mechanical effects (section Well-Mixed Molecular Model of Inflammation and Tissue Remodeling). This is followed by an AB molecular model for inflammation and remodeling coupled with a FE model of biomechanical tissue that supersedes those limitations (section Agent Based Model of Inflammation and Coupling to the Finite Element Model).

### Well-mixed molecular model of inflammation and tissue remodeling

In order to prepare the implementation of the AB model and define the rules thereof, we performed a large bibliographical study to obtain relevant information about:

- cytokines IL_1_, IL_8_, IL_10_, TNFα and TGFβ production,
- macrophage migration, activation and differentiation into M_1_ and M_2_ types,
- feedback loops in the production of pro- and anti-inflammatory cytokines
- the role of MMPs on collagen cleavage and fibroblast deposition which are important terms for elastin degradation and remodeling.

This literature (Ignotz and Massagué, 1986; Onozaki et al., 1988; Oliver et al., 1993; Bellingan et al., 1996; Tsutsumi et al., 1996; Darby et al., 1997; Meng and Lowell, 1997; Hehenberger et al., 1998; Horio et al., 1998; Cobbold and Sherratt, 2000; Steinmüller et al., 2000; Eberhardt et al., 2002; Huang et al., 2002; Maass et al., 2002; Zhang et al., 2003; Mantovani et al., 2004; Porcheray et al., 2005; Tanaka et al., 2005; Edwards et al., 2006; Lenga et al., 2008; Marino et al., 2008; Moro et al., 2008; Jin and Lindsey, 2010; Wang et al., 2012) is reported in Reference section and it is associated to the different biological parameteres considered in Table 1. We focus mainly on the well-vetted interactions between different types of macrophages, pro- and anti-inflammatory cytokines, fibroblasts, collagen deposition and degradation, neutrophils and elastase production. Those interactions were already described by Brown et al. (2011), Jin et al. (2011), and Wang et al. (2012) and our main contribution was to integrate all the available information of the different biological processes and adapt them for the specific case of emphysema modeling. The final model we used is composed by two algebraic equations and thirteen coupled non-linear ordinary differential equations (ODE). This model belongs to the category of well-mixed (WM) systems in the sense that no spatial effects are considered.

**Table 1:** Parameters of the AB model.

**S**	**References + biological meaning**	**Value**
*k_*m*12_*	[est] Transition rate M_1_-M_2_	0.075 day^−1^
*k_2_*	(Porcheray et al., 2005) Activation rate of IL_1_ for M_1_	0.1 ml/pg/day
*k_3_*	(Porcheray et al., 2005) Activation rate of TNF_α_ for M_1_	1 ml/pg/day
*k_4_*	(Porcheray et al., 2005) Activation rate of IL_10_ for M_2_	0.3 ml/pg/day
*k_5_*	(Edwards et al., 2006) Secretion rate of IL_10_ by M_2_	5e^−4^ pg/cell/day
*k_6_*	(Wang et al., 2012) Secretion rate of TNF_a_ by M_1_	7e^−4^ pg/cell/day
*k_7_*	(Meng and Lowell, 1997; Mantovani et al., 2004) Secretion rate of IL_1_ by M_1_	5e^−4^ pg/cell/day
*k_8_*	(Huang et al., 2002) Secretion rate of TGFβ by M_2_	0.07 pg/cell/day
*k_9_*	(Cobbold and Sherratt, 2000) Secretion rate of TGFβ by F	0.04 pg/cell/day
*k_10_*	(Hehenberger et al., 1998) Fibroblast growth rate	0.924 cell/day
*k_11_*	(Ignotz and Massagué, 1986) Collagen deposition rate by F	20 μg/cell/day
*k_12_*	[est] Secretion rate of MP_9_ by M_1_	3 pg/cell/day
*k_13_*	[est] Secretion rate of IL_8_ by M2	5e^−4^ pg/cell/day
*k_14_*	[est] Recruit. of neutrophils by IL8	8 pg/ml
*k_15_*	[est] Secretion rate of elastase by N	3 pg/cell/day
*k_*m*21_*	(Steinmüller et al., 2000) Transition rate M_2_-M_1_	0.05 day^−1^
μ	(Bellingan et al., 1996) Macrophage emigration rate	0.2 day^−1^
μ_N_	[est] Neutrophils emigration rate	day^−1^
c_IL1_	(Onozaki et al., 1988) IL1 promotion on M_1_	10 pg/ml
c_Tα_	(Onozaki et al., 1988) TNF_a_ promotion on M_1_	10 pg/ml
c_IL10_	(Onozaki et al., 1988) IL10 promotion on M_2_	5 pg/ml
c_1_	(Wang et al., 2012) IL_10_ inhibition on IL_10_	100 pg/ml
c	(Marino et al., 2008) IL_10_ inhibition on IL_1_ TNF_α_	25 pg/ml
d_IL10_	(Jin and Lindsey, 2010) Decay rate of IL_10_	2.5 day^−1^
d_Ta_	(Oliver et al., 1993; Tsutsumi et al., 1996) Decay rate of TNF_a_	55 day^−1^
d_IL1_	(Lenga et al., 2008) Decay rate of IL_1_	0.2 day^−1^
d_Tb_	(Zhang et al., 2003) TGF_β_ degradation rate	15 day^−1^
d_FC_	(Darby et al., 1997) Fibroblast apoptosis rate	0.12 day^−1^
d_M_	(Eberhardt et al., 2002; Moro et al., 2008) MMP degradation rate	0.875 day^−1^
λ	(Horio et al., 1998; Maass et al., 2002) Sec. rate TGF_β_ by M_c_	5e^−6^ pg/c/d

*Apart from the indicated sources, also Jin et al. (2011) and Wang et al. (2012)*.

**Figure 1:**
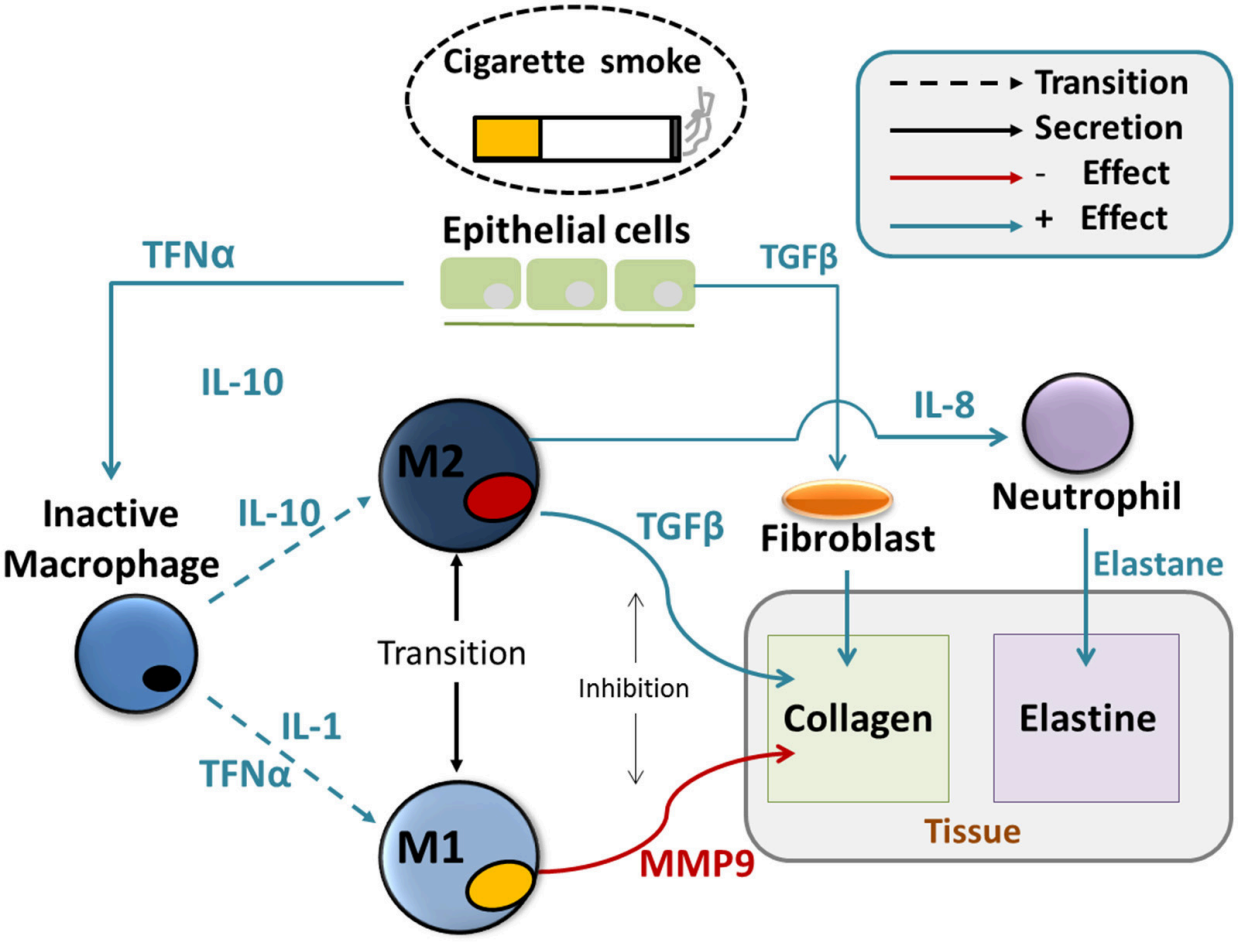
Agent based model of tissue destruction in emphysema progression. Particles coming from inhaled smoke cause secretion of cytokines such as TNFα and TGFβ by the epithelial cells. Those act, at first, as chemotactic factors and attract undifferentiated alveolar macrophages and fibroblasts. and the alveolar macrophages. Secondly, they induce the activation of the macrophages and their differentiation in the M_1_ and M_2_ subtypes. Those will create a delicate dynamical balance between inflammatory and anti-inflammatory signals such as IL_1_, IL_8_, and IL_10_ that affect the activation of protease such as MMPs, the recruitment of neutrophils and fibroblasts. MMPs directly cleavage the collagen from the tissue and are responsible for the deposition of abnormal collage that leads to fibrosis, together with fibroblasts. Elastase destroys the elastin in the tissue. Both abnormal collagen deposition and reduction in elastin deteriorate the mechanical properties of the tissue.

These equations are presented below (Equations 1–15) and the biology they reflect can be schematically represented in an integrated picture of the main molecular and cellular actors that regulate the chronic immune response and the consequent changes in tissue properties (Figure 1), after initial particle deposition on the lung tissue.

The aforementioned particle deposition causes sustained inflammation of the tissues with a fast secretion of Tumor Necrosis Factor alpha (TFNα-Tα in the equations for brevity) and a slow secretion of Transforming Growth Factor beta (TGFβ-Tβ in the equations for brevity) respectively by monocytes (M) and epithelial cells. These cytokines attract monocytes, according to the model proposed by Wahl et al. (1987) (Equation 1):

(1) $$M(T_{\alpha })=0.335T_{\alpha }^{3}\;-6.309T_{\alpha }^{2}+32.281T_{\alpha }+57.302$$

and further govern the differentiation between inactivated macrophages (*M*_*un*_) and the specific sub-types M_1_ and M_2_, according to Equations (2–4):

(2) $$\begin{array}{l} {\dot{M}}_{un}=M(T_{\alpha })-k_{2}M_{un}\frac{IL_{1}}{IL_{1}+c_{IL1}}-k_{3}M_{un}\frac{T_{\alpha }}{T_{\alpha }+c_{T\alpha }}\\ \;-k_{4}M_{un}\frac{IL_{10}}{IL_{10}+c_{IL10}}-\mu M_{un} \end{array}$$

(3) $$\begin{array}{l} {\dot{M}}_{1}=k_{2}M_{un}\frac{IL_{1}}{IL_{1}+c_{IL1}}+k_{3}M_{un}\frac{T_{\alpha }}{T_{\alpha }+c_{T\alpha }}+k_{m21}M_{2}\\ \;-k_{m12}M_{1}-\mu M_{1} \end{array}$$

(4) $${\dot{M}}_{2}=k_{4}M_{un}\frac{IL_{10}}{IL_{10}+c_{IL10}}-k_{m21}M_{2}+k_{m12}M_{1}-\mu M_{2}$$

We see that all attracted monocytes will become inactivated macrophages first, and then switch to one of the two sub-types depending on constants k_2−4_ and the concentration of pro-inflammatory cytokines IL_1_, TFNα and anti-inflammatory cytokine IL_10_. The pro-inflammatory cytokines will promote differentiation to M1 and the anti-inflammatory ones to M2. The promotion effect of the cytokines is mediated by the Hill equation for a cooperative binding type (Stefan and Le Novère, 2013) with coefficients *c*_*IL*1_, *c*_*Tα*_, and *c*_*IL*10_. In time, the transition from *M*_*1*_ to *M*_*2*_ can be reversed, with constants *k*_*m12*_ and *k*_*m21*_. Eventually, the macrophages will be removed through the lymphatic system with rate μ.

Continuing our discussion of Figure 1, we see that each macrophage type will now secrete cytokines with a dynamic expressed by Equations (5–7):

(5) $${\dot{IL}}_{10}=k_{5}M_{2}\frac{c_{1}}{IL_{10}+c_{1}}-d_{IL10}IL_{10}$$

(6) $${\dot{T}}_{\alpha }=k_{6}M_{1}\frac{c_{1}}{IL_{10}+c_{1}}-d_{T\alpha }T_{\alpha }$$

(7) $${\dot{IL}}_{1}=k_{7}M_{1}\frac{c_{1}}{IL_{10}+c_{1}}-d_{IL1}IL_{1}$$

Here we see in Equation (5) that *IL*_*10*_ is secreted by *M*_*2*_ proportionally to k_5_ and regulated by self-inhibition with effectiveness c1. Eventually, it is degraded with half-time decay rate *d*_*IL10*_.

In Equations (6, 7) we have an analogous process for the secretion of TFNα and *IL*_*1*_ by the *M1* macrophages subtype.

Additional TGFβ is secreted from fibroblasts (F) and *M*_*2*_ to increment deposition of collagen in the composition:

(8) $${\dot{T}}_{\beta }=k_{8}M_{2}+k_{9}F-d_{T\beta }T_{\beta }$$

(9) $$F_{g}(T_{\beta })=0.05T_{\beta }^{3}\;-0.98T_{\beta }^{2}+6.54T_{\beta }+7.11$$

(10) $$\dot{F}=k_{10}F_{g}(T_{\beta })F-d_{F}F$$

(11) $$\dot{C}=k_{11}F-d_{FC}MMP\;C$$

(12) $$\dot{MMP}=k_{12}M_{1}-d_{FC}MMP\;C-d_{M}MMP$$

where *K*_*8*_ is the secretion rate by *M*_*2*_, *k*_*9*_ the one by fibroblasts and d_Tb_ the decay rate in Equation (8). Fibroblasts proliferates from the population of already existing cells proportionally to TGF_β_ in Equations (9–10) and emigrate with rate *d*_*f*_. Collagen deposition is governed by Equation (11), where we have to consider the deposition rate *k*_*FC*_, and the degradation effect of matrix-metallo-proteinases (MMP). Those are enzymes produced by *M*_*1*_ that degrade the collagen, as described in Equation (12).

Finally, macrophages attract neutrophils to the wound site by secreting IL_8_, and those release the elastase enzyme that cleaves the elastin bonds in the fibers:

(13) $${\dot{IL}}_{8}=k_{13}M_{2}\frac{c_{2}}{IL_{8}+c_{2}}-d_{IL8}IL_{8}$$

(14) $$\dot{N}=k_{14}(1-\frac{N}{N_{\max}})\frac{IL_{8}}{IL_{8}+c_{IL8}}-\mu_{N}N$$

(15) $$\dot{E}=k_{15}N-d_{E}E$$

IL8 secretion (Equation 13) is similar to Equation (5), with constant *k*_*13*_, a self-inhibition term with efficacy *c*_*2*_ and a degradation constant of *d*_*IL8*_. Equation (14) governs the recruitment of neutrophils up until their maximum value N_max_ with a cooperative effect of *IL*_*8*_ and an emigration rate of μ_*N*_. Finally the density of elastase is dependent upon the number of neutrophils and the inactivation rate, *d*_*E*_.

The final proportion of elastase and collagen density is directly used in our biomechanical model to calculate the properties of the lung tissue for the FEM simulation as discussed at the end of the next section.

All the values for the discussed parameters are presented in Table 1 and the related literature is listed in Reference section.

### Agent based model of inflammation and coupling to the finite element model

In order to add spatial effects to the molecular model of inflammation and tissue remodeling, an AB model is created, using Equations (1–15) as a basis for the behavior of the agents. The first important difference is that the simulation of the agents happens on a grid. This gives the model an inherent spatial aspect and allow us to consider additional details w.r.t. the WB model. For instance, now the composition of the alveolar unit (AU) -which includes among others epithelial cells, collagen, elastin and basement membrane (Zemans et al., 2015)- becomes relevant. In our case, every cell of the grid represents a small portion of the AU with different variables accounting for the content of elastin, collagen, the cytokines and the structural integrity of the cells (called “tissue-life” in the following).

During the simulation a “smoking” signal determines whether we introduce particles into the simulated AU or not. This signal is a periodic square wave with frequency *f*_s_ and intensity *e*_*s*_. The intensity quantifies the *exposure*, that is, the number of particles inhaled in each cycle. The signal starts from zero and last for a total smoking time of T_s_. By varying frequency, intensity and total time, we can study the effect of particles on the model as detailed in experiment of section Experiment to Characterize Parameter Sensitivity. After the end of the total smoking time, the model is allowed to run for some additional time steps in order to reach equilibrium again.

The initial, unperturbed, dynamic of the system includes a small number of inactivated macrophages that move randomly, “patrolling” the tissue and searching for solid particles, similarly to the mononuclear cells behavior described by Auffray et al. (2007). When the smoking signal is active, inhaled particles deposit and cause an initial rapid rise of TFNα that attracts inactive macrophages to the deposition site according to Equation (2). From there, according to the dynamics described in Equations (3–4), macrophages differentiate in M1 or M2 subtypes which respectively govern the production of pro-inflammatory (Equations 6, 7, 12) and anti-inflammatory cytokines (Equations 5, 8, 13).

As previously indicated in Brown et al. (2011), at sites with high levels of pro-inflammatory cytokines, tissue is damaged by a complex network of interconnected factors called Damage-associated Molecular Patterns (DAMPS) (Matzinger, 2002; Lotze et al., 2007). This aspect was not included in the WM model because of its specific spatial nature, but it is implemented in the AB model where the tissue life of the AU is reduced proportionally to the inflammation level. Damaged tissue (i.e., with reduced tissue-life) in turn, start secreting TFGβ to recruit fibroblasts for wound healing as in Equations (9, 10).

The model tracks separately the amount of collagen and elastin in the tissue and their equilibrium varies depending on the concentration of fibroblasts, neutrophils, elastase and MMPs as in Equations (11, 12, 14, 15).

The cellular death caused by DAMPS and the amount of collagen and elastin, all affect the mechanical properties of the tissue used in the FE simulations. In this first version we use an elastic, isotropic material implemented in Elmer FEM software (Råback, 2013). Now, on the one hand, when a cell dies, we reduce its Young's modulus (E_Tissue_) to 1 Pa, to account for the fact that it contributes no more to the elastic properties, but without changing the topology of the mesh. On the other hand, if the cell is not dead, its Young's modulus is calculated as a linear mixture of the corresponding concentration of elastin and collagen as in Equation (16). The initial values are *E*_*el*_ = *0.1 kPa* and *E*_*cl*_ = *20 kPa*, as described by Suki et al. (2011).

(16) $$E_{Tissue}=\delta_{el}c_{el}E_{el}\;+\;\delta_{cl}c_{cl}E_{cl}$$

$$\delta_{el}=0.7;\;\delta_{cl}=\;0.3,\;c_{el}\in [0,1];\;c_{cl}\in [0,1];$$

The material properties are calculated and loaded in the solver as continuous static field using a custom made Fortran code.

Apart from the molecular damages caused by DAMPS, the tissue can also die because it was subjected to too much strain during the mechanical simulations. While elastin withstands deformation as high as 100%, the maximum tensile strain of pure collagen fibrils with low cross-link density is considered to be around 10% of the initial length (Depalle et al., 2015; Sherman et al., 2015). Accordingly, the maximum tensile strain for each cell is calculated weighting the previous values for the amount of elastin and collagen contained.

**Figure 2:**
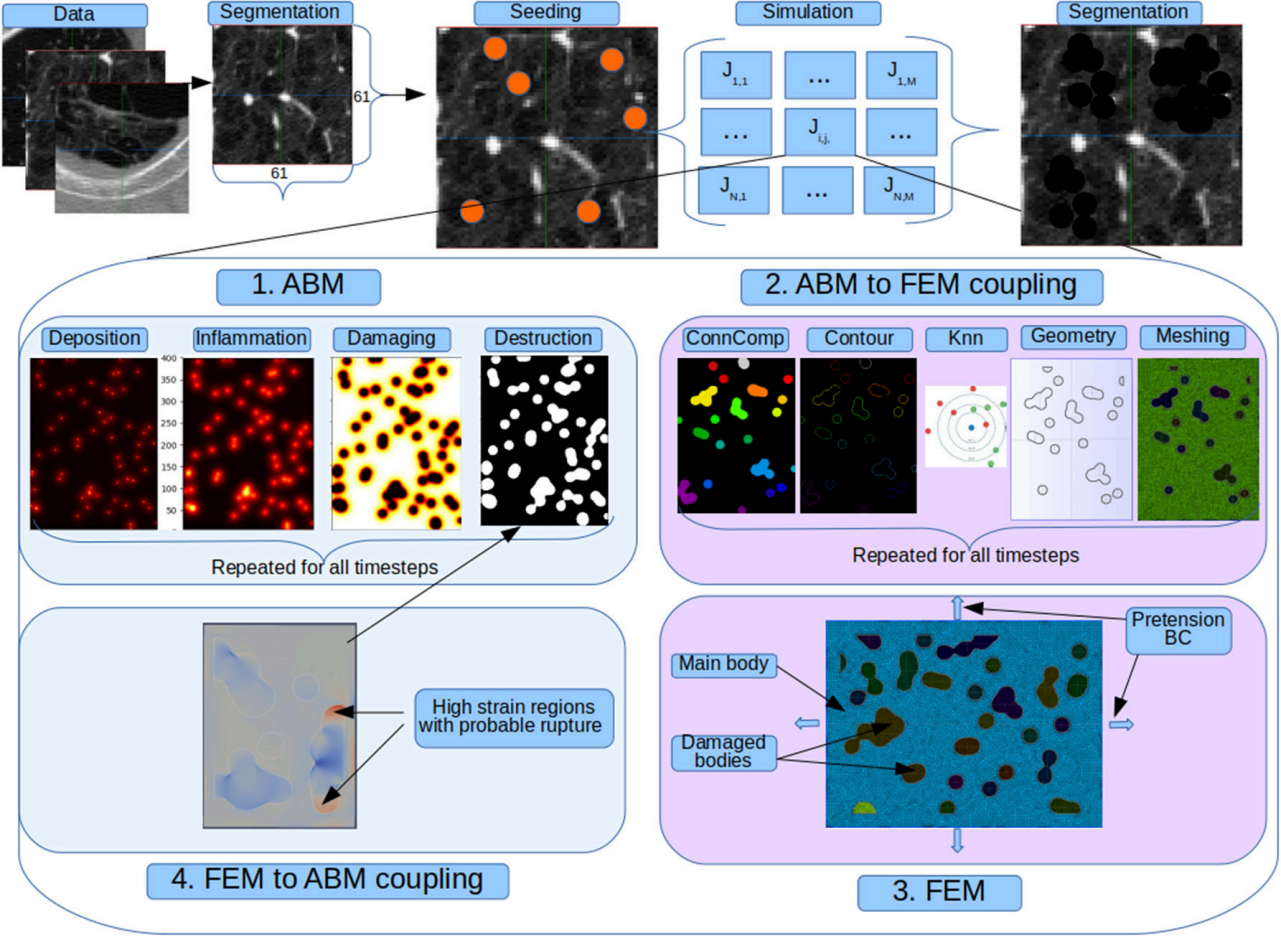
Full coupled model. Original patches from a public emphysema database are segmented to separate the parenchyma from the vessels and airways and seed deposition is simulated. For each seeded pixel and for all its neighbors we run a simulation job that represent the evolution of 130 alveoli. In each job there is a cyclic sequence between the agent and finite element model. At each step the former simulate additional particle deposition that accounts for continued smoking; release of inflammatory cytokines and degradation of mechanical properties. Periodically the AB model is frozen and the calculated tissue properties are imported in the AB-FE coupler code which will reconstruct a topologically equivalent geometry, recover the contours of the damaged zones and assign new material properties taking into account the final amount of collagen and elastin from the AB model. The resulting information is passed to the FE solver that runs until convergence and then export the strain results for further processing. After the FE solver has run, the second coupler code, FE-AB is run to import the strain field and calculate which fibers, if any, have been destroyed in the simulation. It thus updates the AB status and restarts it with the updated state.

We present in Figure 2 the indirect coupling strategy used for the AB and FE models. At each step the former simulate additional particle deposition that accounts for continued smoking; release of inflammatory cytokines and degradation of mechanical properties. Periodically the AB model is frozen and the calculated tissue properties are imported in the AB-FE coupler code which will reconstruct a topologically equivalent geometry, recover the contours of the damaged zones and assign new material properties taking into account the final amount of collagen and elastin from the AB model. The resulting information is passed to the FE solver that runs until convergence and then export the strain results for further processing. After the FE solver has run, the second coupler code, FE-AB is run to import the strain field and calculate which fibers, if any, have been destroyed in the simulation. It thus updates the AB status and restarts it with the updated state.

## Experimental setup

The next sections deal with the more experimental part of our work. First, we explain the inner working of the coupling between AB and FEM solvers (section Procedure to Couple AB and FEM). Then, we present the meshing process (section Mesh Creation and Sensitivity). In the central part of this section we detail the two main experiments that validate our implementation: the first is an initial exploration of the sensitivity of the model to initial parameters (section Experiment to Characterize Parameter Sensitivity), while the second is the validation on a public CT image dataset of emphysematous lungs (section Experiment to Study the Emphysema Progression in Clinical Images). Finally, we briefly discuss the High Performance Computing infrastructure we used to run the studies (section High Performance Computing).

### Procedure to couple AB and FEM

In a typical execution cycle, the AB model is stopped at regular intervals and control is transferred to the FE model for analysis of the mechanical strains. After each interruption of the AB simulation, the latest iteration of this simulation is saved to disk and the AB-FE coupling code first calculates the percentage of damaged tissue area, as predicted by the AB model, and evaluates whether there is enough healthy tissue to proceed with the mechanic simulation. If this is the case, the saved status of the AB model is inspected to retrieve the last topology of the computational grid and the amount of collagen and elastin is used to calculate the new Young modules of the tissue according to Equation (16). This information is used together with connected component analysis, morphological operators and k-Nearest Neighbors (kNN) classifiers, to extract the contours of the broken tissue, define a 2D mesh and assign mechanical properties to each element. Materials, boundary conditions and solver parameters are adjusted if necessary and a case directory is created for the FE solver. The FE model runs asynchronously until convergence of the steady state and deformation and displacement fields are saved in a vtk compatible format (*vtu*). After that, the FE-AB coupling code is executed again. It reads back the strain fields from the solver status files and determines which, if any, nodes of the mesh have exceeded their maximum strain. Those are added to the damaged zone and the agent simulation is restarted. Cycle by cycle the coupled simulations continue until tissue damage is above 80% of the area or until the desired simulated time is reached.

**Figure 3:**
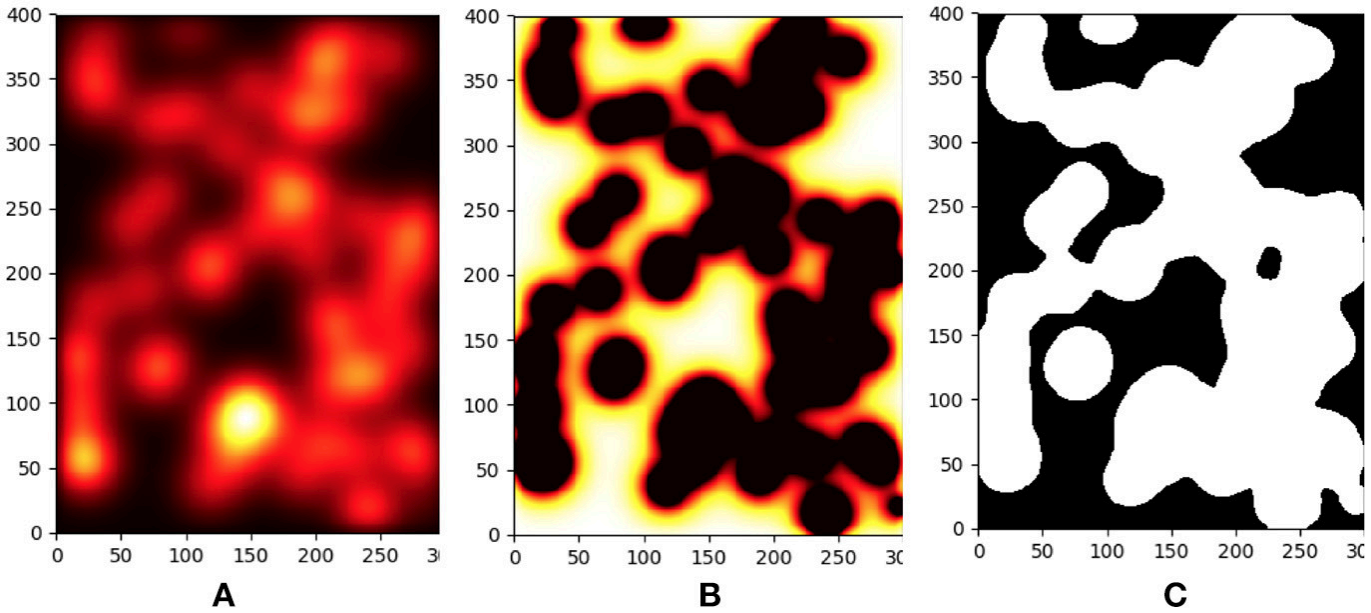
Progression of parenchyma destruction in Agent Based Model. From left to right we see the effect of increased inflammation, tissue damage and final destruction. **(A)** Concentration of proteases in a small sample of the tissue during model execution. **(B)** Due to the continued effect of the high proteases levels the tissue is damaged. **(C)** Snapshot of the damaged tissue as sent to the FEM model. Tissue in foreground (white) has greatly diminished mechanical properties.

**Figure 4:**
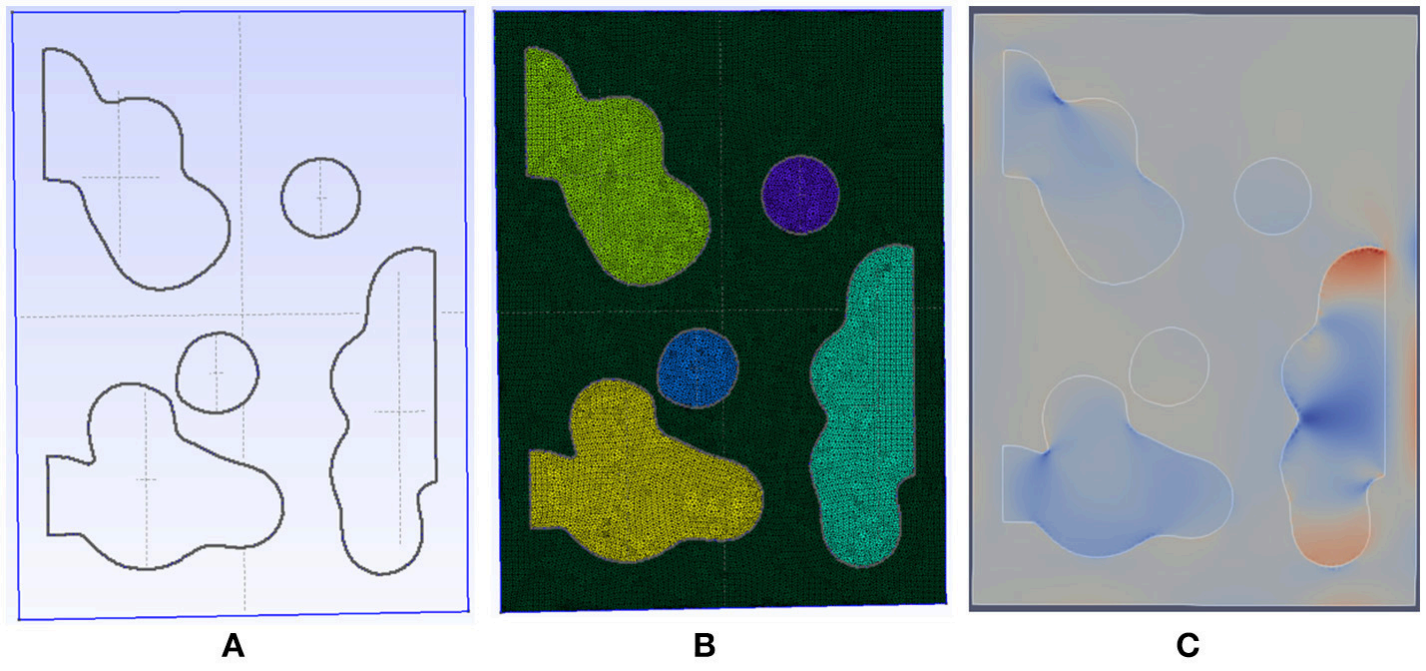
Detail of the meshing process for a patch with initial emphysema formation. **(A)** The geometry is automatically generated and meshed by gmsh using our code taking into account Agent Based Model. **(B)** The resulting mesh has an adaptive size to ensure fast convergence and is able to capture complex shape for the destroyed tissue. **(C)** The generated mesh is then connected to our FEM solver and simulated until convergence is reached. In this image, the displacement field is shown.

A detailed view of typical results for the inflammation, meshing and mechanical process is shown in Figures 3, 4.

### Mesh creation and sensitivity

We use 2D FE meshes with topologies equivalent to the AB simulation grids. The exact size and topology of each mesh is thus very dependent on the current state of the simulation. In addition, an optimization step is run after the first mesh creation using Gmsh^2^. The average mesh contains around 50,000 polygons with four nodes. We manually refined the parameters of the mesh creation to ensure a quick convergence of the FE simulations, while keeping a low computational cost, necessary to ensure reasonably fast and smooth interactions between the AB and the FE models.

### Experiment to characterize parameter sensitivity

We studied several possible parameters to characterize the model's behavior. First, we varied the quantity of particles and the frequency with which they are added. We varied the number of particles inhaled in each smoking step from 0 to 20 and the smoking time from 10 to 90 simulation steps, for a total of 25 experiments. This will be referred to as the “*exposure”* experiment in the results section.

In order to asses the sensitivity to the parameters, we selected and varied six main parameters of the model as described in Table 2. This resulted in a total of 2^6−2^ = 16 experiments following a fractional factorial analysis. We will refer to this as the “*parameters*” experiment in the results section.

**Table 2:** Parameters experiments.

**Experiment**	**K_8_ (pg/cell/day)**	**dIL_10_ (2.5 day^−1^)**	**K_12_ (pg/cell/day)**	**K_13_ (10^−3^) (pg/cell/day)**	**d_FC_ (day^−1^)**	**d_M_ (day^−1^)**
1	0.7	2.5	3	0.5	0.12	0.875
2	0.14	2.5	3	0.5	0.24	0.875
3	0.7	5	3	0.5	0.24	1.75
4	0.14	5	3	0.5	0.12	1.75
5	0.7	2.5	6	0.5	0.24	1.75
6	0.14	2.5	6	0.5	0.12	1.75
7	0.7	5	6	0.5	0.12	0.875
8	0.14	5	6	0.5	0.24	0.875
9	0.7	2.5	3	1	0.12	1.75
10	0.14	2.5	3	1	0.24	1.75
11	0.7	5	3	1	0.24	0.875
12	0.14	5	3	1	0.12	0.875
13	0.7	2.5	6	1	0.24	0.875
14	0.14	2.5	6	1	0.12	0.875
15	0.7	5	6	1	0.12	1.75
16	0.14	5	6	1	0.24	1.75

*Values of the parameters used in the 2^6−2^ = 16 experiments*.

### Experiment to study the emphysema progression in clinical images

One of the main objective of this model was to predict the development of emphysema in time. We devised an initial way to test our hypothesis using a public lung image dataset. We explain our approach in the following sections.

#### Dataset

We test our system against the public CT Emphysema database (Sorensen et al., 2010). We use 168 square patches manually annotated in a subset of the 115 high-resolution CT (HRCT) slices. As explained in the previous reference, CT scanning was performed using General Electric (GE) equipment (LightSpeed QX/i; GE Medical Systems, Milwaukee, WI, USA) with four detector rows. The acquisition protocol was: in-plane resolution 0.78 × 0.78 mm, slice thickness 1.25 mm, tube voltage 140 kV, and tube current 200 mAs. The slices were reconstructed by using a high-spatial-resolution (bone) algorithm. The data comes from a study group of 39 subjects, including 9 never-smokers, 10 smokers, and 20 smokers with COPD. Figure 2 shows a sample of each of the three categories of images.

### Pre-processing

All slices were automatically segmented and reviewed to create a mask of only parenchyma tissue. In order to prepare the computational model, we first segmented the pulmonary tissue in the lung patches, using a fixed threshold of −750 HU. Stereological analysis of the lung parenchyma revealed a mean of 500 million alveoli per double lung in the normal population, with a mean alveolar volume of around 4.2 × 10^6^ μm^3^ and, on average, 170 alveoli per cubic millimeter (Ochs et al., 2004). In our case, with an anisotropic spacing of 0.78 × 0.78 × 1.25 mm^3^, this corresponds to roughly 130 alveoli per voxel. For each voxel of this binary mask we generate a planar grid of the 130 alveoli that is used as a computational mesh.

#### Particle deposition and simulation

As detailed in Figure 2, once the patch has been segmented, random pixels of the parenchyma and their neighbors are marked as “affected” and, for each one, a new simulation of the AB and FE models is run. Final results are mapped back into the main image patch and the updated mechanical properties calculated by the coupled AB-FE model are linearly translated back into HU values. In this way, we simulate the typical darkening of the CT scan caused by emphysema progression.

### High performance computing

Among many different frameworks available for AB modeling (Abar et al., 2017), we chose to use Pandora (Rubio-Campillo, 2014), for its ease of programming and superb scalability. The model is implemented in an in-house version, specifically modified to allow biological model developments and available online^3^.

In order to satisfy the high demand in computational resources, we run the simulations on our institution's supercomputing SNOW Linux cluster. The cluster is currently composed by 20 computing nodes and a total of 840 cores with a theoretical calculation capacity of 8.49 Tflops. Highly relevant for agents simulations were six GeForce GTX TITAN X GPU with 12 Gb of memory.

## Results and discussion

In this section we present the results of the two main experiments that we have used to validate our implementation. Those experiments were previously discussed in detail respectively in sections Experiment to Characterize Parameter Sensitivity and Experiment to Study the Emphysema Progression in Clinical Images.

### Parameter sensitivity and model analysis

**Figure 5:**
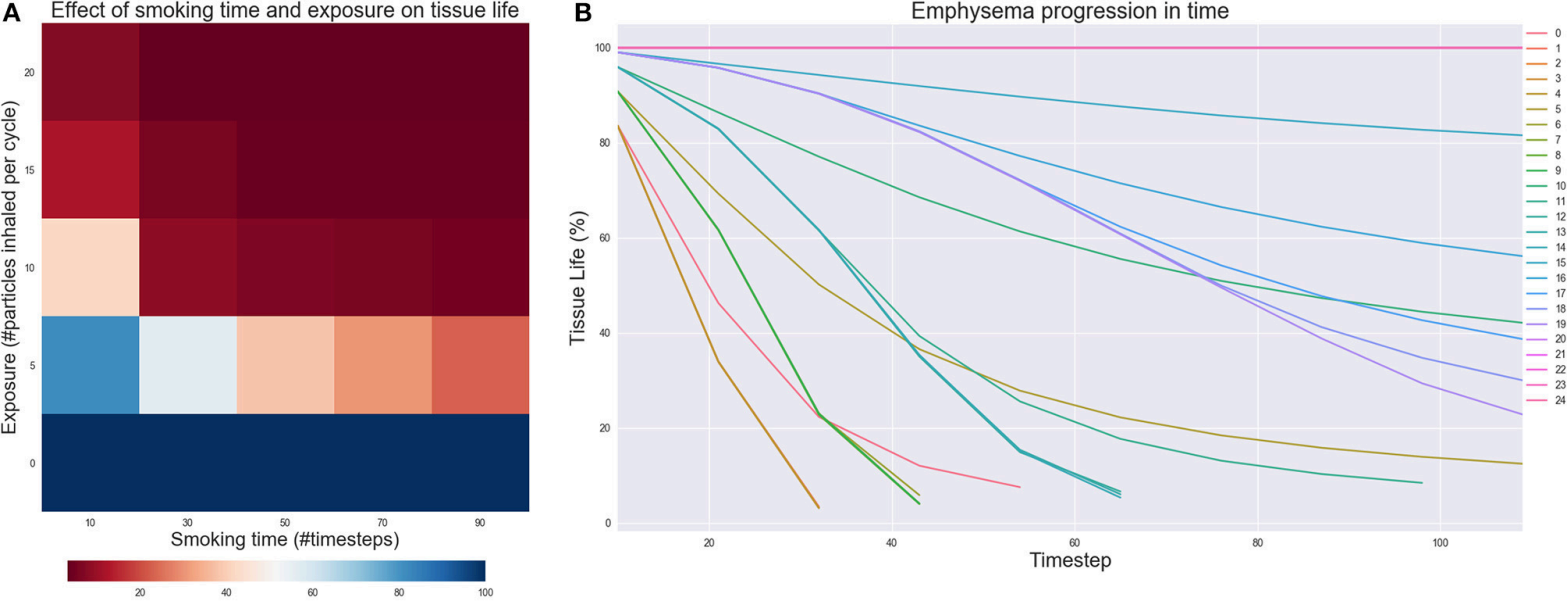
**(A)** Effect of changing the number of particles inhaled for each simulated smoking (exposure) and the total time of the simulation spent smoking (Smoking time). The value of each cell is the residual tissue life in % after the simulation stopped. **(B)** Emphysema progression in time. The parameters of the experiments are in figure **(A)**: starting from experiment 0 in the lowest left corner to experiment 24 in the upper right.

The results of the *Exposure* experiment are shown in Figures 5A,B. Figure 5A illustrates the effect of changing the number of particles inhaled for each simulated smoking exposure and the total time spent smoking. When exposure is zero, the model is able to capture that the tissue should remain healthy no matter how long the simulation runs. However, as both the exposure and total time spent smoking increase, the tissue starts getting damaged, independently on the values of the rate constants. For lower to medium exposures, the implicit stochasticity of the AB model and the variability of the rate constants lead to some the fluctuations of the results in function of the smoking time, but tissue life is always reduced by at least 50%. For higher exposure, tissue damage is irreversible and continues even after smoking cessation is simulated, as shown in Figure 5B. These outcomes nicely reflect the fact that smoke frequency and exposure are considered as one of the main risk factors for the development of COPD and emphysema (Yoshida and Tuder, 2007; Liu et al., 2008).

In the model, the mechanical damage largely depends on the regulation of the collagen content, because the stiffness of this macromolecule is two orders of magnitude the stiffness of elastin. The degradation of collagen is heavily affected by TNFα through the recruitment of monocytes (Equation 1) and the activation of macrophages into M1 type (Equation 3) with positive feedback loops generated through IL1 (Equations 3, 7) and TNFα (Equation 6). In contrast, the activation of macrophages into M2 type is promoted by the anti-inflammatory cytokine IL10 (Equation 4), the production rate of which is positively retro-alimented by M2 macrophages (Equation 5). While IL10 inhibits TNF-alpha and IL1 (Equations 6, 7), it is also self-inhibited (Equation 5). Hence, the anti-inflammatory effect of IL10 is limited compared to the strong inflammatory effects of TNFα and IL1, because less positive feedback in favor of the promotion of type M2 activated macrophages. In the *Parameters* experiment, the exposure and total smoking time parameters were set to respectively 10 particles and 50 time steps to ensure that the system would be in a medium damage situation. Results were all very similar and the tissue life in each time step only varied with an average standard deviation of 0.166 units, revealing that the above interpretation of the model holds true regardless the variation of the rate constants within the considered ranges of values.

According to the analysis of the model equations, the promotion of the anabolic TGFβ (Equation 8) should be limited compared to the promotion of the catabolic MMP (Equation 12), and the persistent inflammation induced by particles should promote the unequivocate destruction of collagen (Equation 11). Nevertheless, we sometimes saw an increase in the mean amount of collagen. This outcome can be due to the mechanical feedback and mechanical tissue damage that promotes the secretion of TGF-beta and provides additional weight to collagen anabolism (Equations 9, 10). In our model emphysema progression, was indeed related to sustained inflammation that continued after smoking cessation (Willemse et al., 2005), but required the additional effect of DAMPS to relate inflammation, altered tissue turnover and tissue mechanics to cell endothelial death. This phenomenon needs to be further explored in a more mechanistic way, but our approximation of DAMPS effects allows qualitative validation of the simulated mechanisms for emphysema progression against clinical data (see below).

### Emphysema progression in clinical images

**Figure 6:**
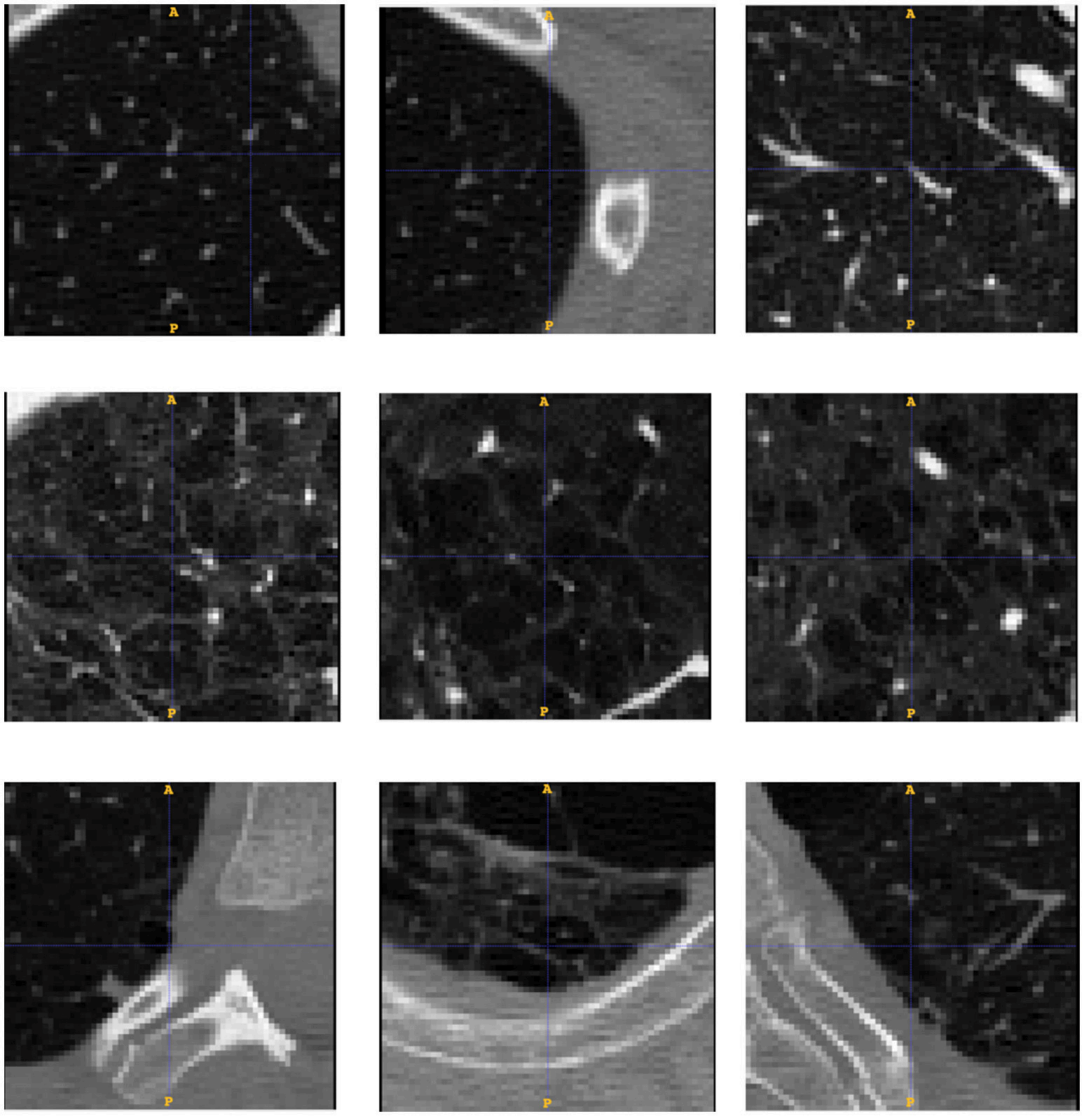
Database patches samples for the three categories of low **(up)**, medium **(middle)** and severe **(down)** emphysema affectation. We see how the affectation is related to the appearance of bigger cluster of low attenuation areas from top to bottom.

To test whether our model is able to produce images similar to those seen by clinicians, we use the public emphysema database described in section Experiment to Study the Emphysema Progression in Clinical Images. Images of some of the patches representative of the data we used in the experiment are presented in Figure 6. Parenchyma destruction in emphysema is strongly associated with decreased HU absorption value in CT images, and many image descriptors are commonly used to (semi-)automatically detect emphysema progression in CT images (Stern and Frank, 1994; Gevenois and Yernault, 1995; Madani et al., 2006). In the present study, emphysema progression is quantified through the well-known Mean Lung Density (MLD) (Heremans et al., 1992).

**Figure 7:**
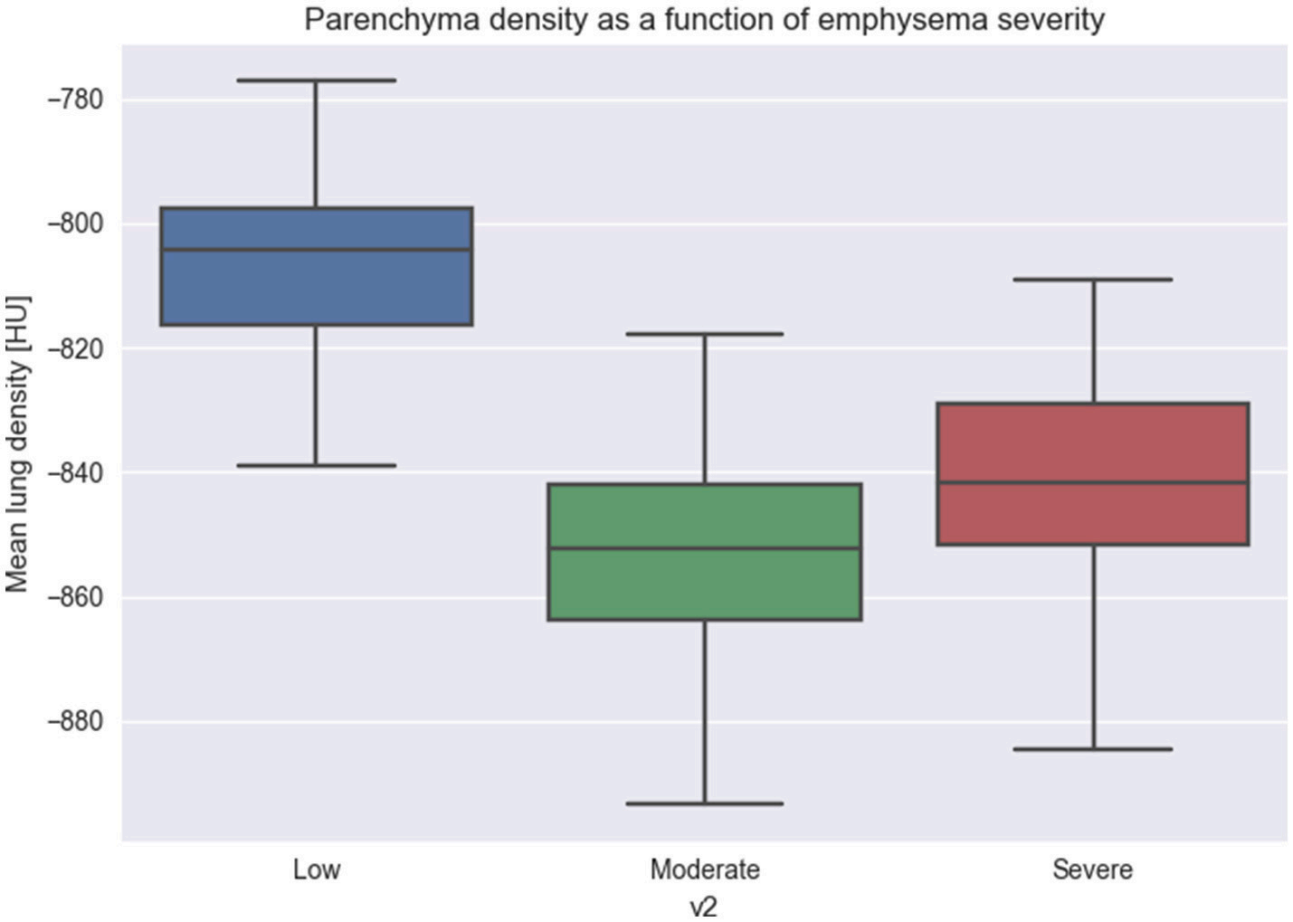
Relation between Mean Lung Density and emphysema progression. We used Mean Lung Density (MLD) to quantify patches belonging to the three emphysema classification levels. The figure shows that emphysema progression is associated with a mean lowering of the MLD values, due to the destruction of parenchyma and the diminishing of the CT attenuation value.

We quantify all the patches from the database and group by the different degrees of emphysema severity. As it can be seen from Figure 7, images with increasing emphysema severity have also a lower MLD score. Differences of more than 40–60 HU between groups (1) and (2), (3) are significant with *p*-value of less than 0.01.

Once the association between emphysema progression and MLD score is determined, we take all the 69 patches annotated with low or no emphysema affectation and use them as input for our model. Images are quantified with MLD before and after model execution and the results are tested with *t*-test for statistical significance of the differences.

**Figure 8:**
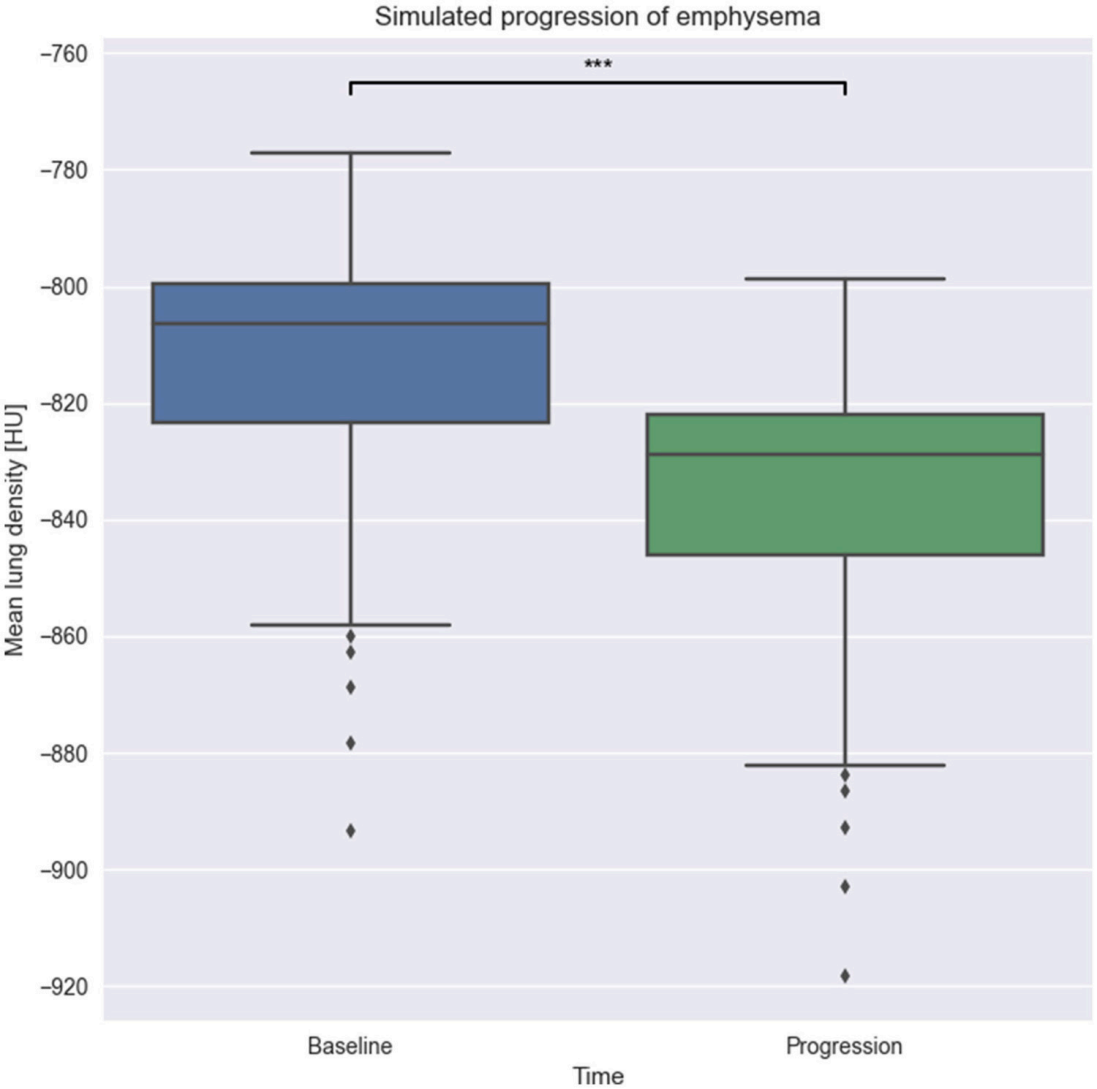
Effect of model execution. We run our emphysema progression model on 69 patches with low emphysema until completion. As expected, the resulting patches have a lower MLD than the original one, showing parenchyma destruction during model run. ***p-value < 0.001.

As we can see in Figure 8, there is a statistically significant difference of about 30 HU between the *baseline* and *progression* groups with a *p*-value of less than 0.001. We thus conclude that our implemented model is able to simulate changes that are in agreement with the progression of emphysema in clinical images quantified by MLD.

### Scaling

The parallelization strategy for the AB part consists in assigning a job for each patch, as they were completely independent from each other, then recursively create a new job per voxel, which is the smaller unit we can parallelize for now. With 69 patches to process and 200 seeds per patch plus their four closest neighbors, this resulted in 69,000 jobs. The AB code uses OpenMP to parallelize the execution of the agents. For the FE part, we use the MPI capability of Elmer solver to partition the mesh and distribute the computation on 5 MPI processes per job. Finally, the coupling between AB and FE is executed in a sequential way with a python script. Most of the code executions for the coupling use libraries for which C code bindings were available (numpy, scipy, and skimage) and, thus, simulations run at almost native speed. During a single job execution, the computational time is taken mostly by the AB model (54%), then by the FE solver (37%) and finally by the coupling part (9%). Each job took about 40 min on the cluster and 1 h on a workstation computer (Intel i7 with 32 GB of RAM). A significant amount of time of the coupled simulations was spent on writing the files to disk to share data between the solvers. This could be reduced in future works by using faster SSD disk or in-memory access. All jobs would have taken months to be processed sequentially on the abovementioned workstation, but required 8 days on our cluster, by using a maximum of 256 simultaneous jobs. The use of the HPC resulted, therefore, in several orders of magnitude of computational time reduction, making the present study actually feasible. All jobs in the cluster use Sun Grid engine.

## Conclusions and future works

In this paper we conceived, developed and tested a high performance multi-scale agent-based model of lung parenchyma evolution after repeated exposure to solid irritants such as the particles that arrive to the lung while smoking. We modeled the simplified behavior of immune system cells such as alveolar macrophages and neutrophils, and also cells in charge of wound healing mechanisms such as fibroblasts. Finally, the tissue behavior under the forces present in the lung during respiration was modeled using a FE elastic model. An initial analysis of sensitivity of the model to parameter variations confirmed (i) the ability of the model to point out particle inhalations as a major risk factor in emphysema pathogenesis, and (ii) the strong inertia of the catabolic shift of cell activity due to sustained inflammation that resulted in sustained damage to most of the tissue. A preliminary validation of the capacity of the model to cause a significant change was performed against clinical images on 69 cases of a public database of CT images affected by emphysema progression.

To the best of our knowledge, this model advances the state of the art because: (1) it includes a more detailed molecular model of inflammation and tissue remodeling (2) uses a FE solver to calculate the response to mechanical solicitations thus allowing for future extensions where arbitrary complex tissue constitutive equations could be used. (3) has a bi-directional coupling between AB and FE models (4) exploits HPC technologies so that enough tissue can be simulated to start validating against imaging data (5) uses clinical CT images to perform an initial validation of the capacity of the model. The implementation of a system of coupled ODEs into AB has the great advantage over a well-mixed model to take into account the spatial aspect, and the formation of self-sustaining spatial patterns that affect substantially the equilibrium points of the system (Brown et al., 2011).

The present model has, of course, several limitations. Simplifications were still made in the immune response and the mechanical model. In particular, the relative importance of DAMPS in the validated model suggest that more mechanistic development of this biological phenomenon are necessary. Additionally, while in this implementation of the model we used a 2D mapping between the alveolar exchange surface and the computational grid, in following works we will explore the effect of extending the connectivity of the tissue to 3D. On top of that, we do not account for heterogeneous tissue structures such as airways or blood vessels. However, we plan to do so in a following extension as the relevant information is already present in the CT images used to initialize the model. Effect of the mesh size and topology should be further explored. In a follow-up study we plan to automatically find the best parameters and better characterize the impact of the mesh on the stability of the solution with a convergence study.

Also, the validation is still somehow limited, as no histological comparison with *ex-vivo* animal models could be performed and the one on CT clinical images is limited to one clinical descriptor, namely the MLD score. As a future work, we are planning a retrospective study with COPD patients with 1-year follow-up. Of course, the real ground truth should be histology, which is unfortunately very difficult to obtain in human subjects. A promising alternative is to use mice models of emphysema.

We suggest that such a model, once properly extended and calibrated with histological and clinical data, could be useful to improve patient classification and prediction of exacerbations and thus contribute to the selection of a personalized therapy.

## Author contributions

MC: conceived the research, designed the model, generated and analyzed the experimental data, validated the implementation and wrote the paper; AO: implemented part of the ABM rules and helped with the collection and analysis of the experimental data; JN: helped with the preparation of the FE model with the writing of the paper and the analysis of the results; MG: oversaw the project and revised the paper. All authors read and approved the final version of the manuscript.

### Conflict of interest statement

The authors declare that the research was conducted in the absence of any commercial or financial relationships that could be construed as a potential conflict of interest.

## Footnotes

^1^INSPIRE - Personalized computational models of COPD progression for patient phenotyping. FIS2017-89535-C2-2-R

^2^Open source: gmsh.info

^3^https://bitbucket.org/mrceresa/pandora
